# ﻿Two new freshwater species of *Surirella* (Bacillariophyta) from the Wuling Mountains, China

**DOI:** 10.3897/phytokeys.201.79626

**Published:** 2022-06-20

**Authors:** Ji-Yan Long, John Patrick Kociolek, David M. Williams, Bing Liu, Wen-Hui Mo, Jin-Hua Chen

**Affiliations:** 1 College of Biology and Environmental Sciences, Jishou University, Jishou 416000, China Jishou University Jishou China; 2 Museum of Natural History and Department of Ecology and Evolutionary Biology, University of Colorado, Boulder, Colorado-80309, USA University of Colorado Boulder United States of America; 3 Department of Life Sciences, the Natural History Museum, Cromwell Road, London SW7 5BD, UK Natural History Museum London United Kingdom

**Keywords:** Helictoglossa-like process, new species, *Surirella*, twisted frustule, ultrastructure

## Abstract

Two sympatric *Surirella* species found at the same specific locality in the Wuling Mountains of China are documented with light and scanning electron microscope. Both species are new to science and named *S.wufluminensis***sp. nov.** and *S.suiningensis***sp. nov.***Surirellawufluminensis* has large frustules that are either clockwise or counterclockwise twisted when viewed with the light microscope, and possesses distinctive fibulae, mound-like outgrowths on the valve surface throughout, raised longitudinal ridges on both sides of the raphe, and two helictoglossa-like processes at one apex internally. *Surirellasuiningensis* has narrowly ovate valve outline, distinctive fibulae, troughs alternating with crests from pole to pole, and two helictoglossa-like processes at one apex internally. These two species do not produce costae on the valve surface in contrast to many species in *Surirella*. This study provides a further two examples of the wide range of morphological diversity in the genus *Surirella*.

## ﻿Introduction

The diatom genus *Surirella*Turpin (1828) includes numerous taxa commonly found in benthic habitats (Hustedt 1930; Krammer and Lange-Bertalot 1991; but see also Hustedt 1942 for a consideration of species from large lakes that may be planktonic). Formal morphological (Ruck and Kociolek 2004) and molecular (Ruck et al. 2016) analyses suggested that the former diagnosing features of *Surirella* and closely-related genera were not supported and that some species of *Surirella*, including its generitype *S.striatula* Turpin and the Pinnatae groups of *Surirella*, are more closely related to some species previously included in *Campylodiscus* C.G. Ehrenberg ex Kützing than they are to other species previously included in *Surirella* (the Fastuosoid group) (Ruck and Kociolek 2004; Ruck et al. 2016).

Wang (2018) considered the surirelloid diatoms from inland habitats of China and recognized 47 different taxa (including only two new species) within Surirellaceae: nine taxa in *Cymatopleura* W. Smith, 29 taxa in *Surirella*, four taxa in *Stenipterobia* Brébisson ex Van Heurck, and five taxa in *Campylodiscus* Ehrenberg ex Kützing. Kociolek et al. (2020) listed 33 freshwater *Surirella* taxa described from China before 2000, including those described by Mereschkowsky (1906) and Skvortzov (1927; 1929a, b, c; 1930, 1938, 1976). Post-2000 until 2019 another 3 new taxa were described (Kulikovskiy et al. 2012; Liu et al. 2019). Among the above 36 taxa listed by Kociolek et al. (2020), only three species, *Cymatopleuraxinjiangiana* Q-M You & J. P. Kociolek, *C.aquastudia* Q-M You & J. P. Kociolek, *Surirellatientsinensis* Skvortzov, were also mentioned in Wang (2018). And most recently Liu et al. (2021) described a new species from China and included the record of another species in the flora of the country.

There are a few taxa in *Surirella* sensu stricto possesseing ‘twisted’ frustules, such as *S.aquastudia* (Kociolek & Q. You) Kociolek, *S.xinjiangiana* (Q. You & Kociolek) Kociolek, and *S.dongtingensis* Bing Liu & Ector (You et al. 2017; Liu et al. 2021). They often appear to different visual discrepancies due to the degree of twist or their position relative to the observer. There are also a few taxa in *Surirella* sensu stricto without costa-stria bundles (sensu Liu et al. 2019), such as *S.stalagma* M.H. Hohn & Hellerman and *S.atomus* Hustedt (English 2011). In this study, we describe two new species belonging to *Surirella* sensu stricto characterized by twisted frustules and the valves lacking costae (thickened siliceous ribs).

## ﻿Materials and methods

The study site is at the course of Wu River running through Suining County, located in the Wuling Mountains of China under a sub-tropical to warm temperate type climate. At the sampling site, epilithic algae were collected from numerous submerged stones showing yellow-brown surfaces indicating the presence of diatoms. Each stone was placed on a plastic plate and its surface was brushed using a toothbrush, with the brushed-off diatom samples being washed into the plate. The diatom samples were transferred into a 100 mL sampling bottles and fixed with 70% ethanol. Two samples were collected from each site. Together with the sample collection, temperature, pH, and conductivity were measured *in situ* with a portable multimeter (HQ40D, HACH Company)—details are presented below in the ‘Distribution and ecology’ section of the species description.

Specimens for permanent slides were air-dried onto coverslips then mounted onto microscope slides using Naphrax. The slides were examined and specimens photographed using a Leica DM3000 light microscope (LM) at ×1000 magnification (objective NA 1.25) and a Leica MC190 HD digital camera. The holotype slides are deposited in the Natural History Museum, London, United Kingdom (BM) and isotypes slides are kept in the Herbarium of Jishou University, Hunan, People’s Republic of China (JIU). For scanning electron microscopy (SEM) observation several drops of the selected cleaned diatom material were air-dried onto glass coverslips that were then attached to aluminium stubs using double-sided conductive carbon strips and sputter-coated with platinum for 20 seconds (Cressington Sputter Coater 108auto, Ted Pella, Inc.). Samples were examined and imaged using a field emission scanning electron microscopy Sigma HD (Carl Zeiss Microscopy) available at Huaihua University, China.

Terminology related to valve morphology follows Ruck and Kociolek (2004) and Van de Vijver et al. (2013), and costa-stria bundle (CSB), over-fibula costa (OFC) follows Liu et al. (2019).

## ﻿Results

### ﻿Class Bacillariophyceae Haeckel

**Order Surirellales D.G. Mann**

**Family Surirellaceae Kützing**

#### Genus *Surirella* Turpin

##### ﻿ Surirella wufluminensis

Taxon classificationPlantaeSurirellalesSurirellaceae

Bing Liu & Kociolek sp. nov.

9FE5A347-FE10-5797-A31A-A0D7C8D6F211

Figs 1 , 2 , 3 , 4

###### Holotype.

**Figure 1.**
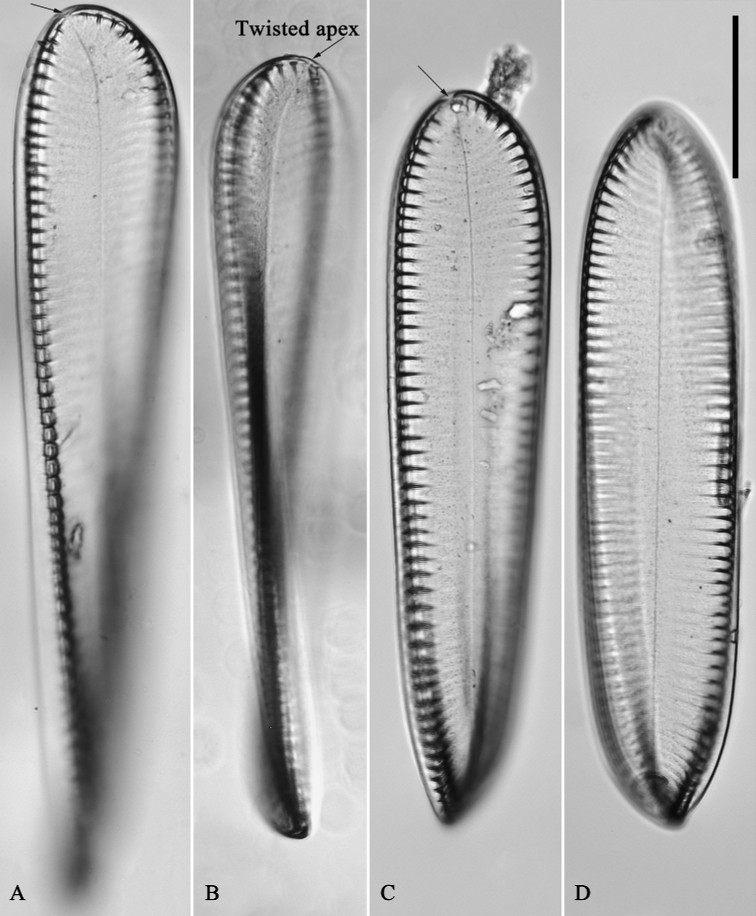
*Surirellawufluminensis* sp. nov., LM, ×400 **A, C** two counter-clockwise twisted valves **B, D** two clockwise twisted valves. Micrograph of the holotype specimen (Fig. A) and isotype specimen (Fig. D). Scale bar: 50 μm.

***Holotype***BM! 81892, specimen circled on slide, illustrated as Fig. 1D; isotype JIU! G202106, specimen circled on slide, illustrated as Fig. 1C.

###### Type locality.

China. Hunan province: the course of Wu River, a sampling point at Changpu Town, Suining County, 26°34.59'N, 110°09.19'E, 300 m a.s.l., collected by Bing Liu, March 22, 2021.

###### Description.

**Figure 2.**
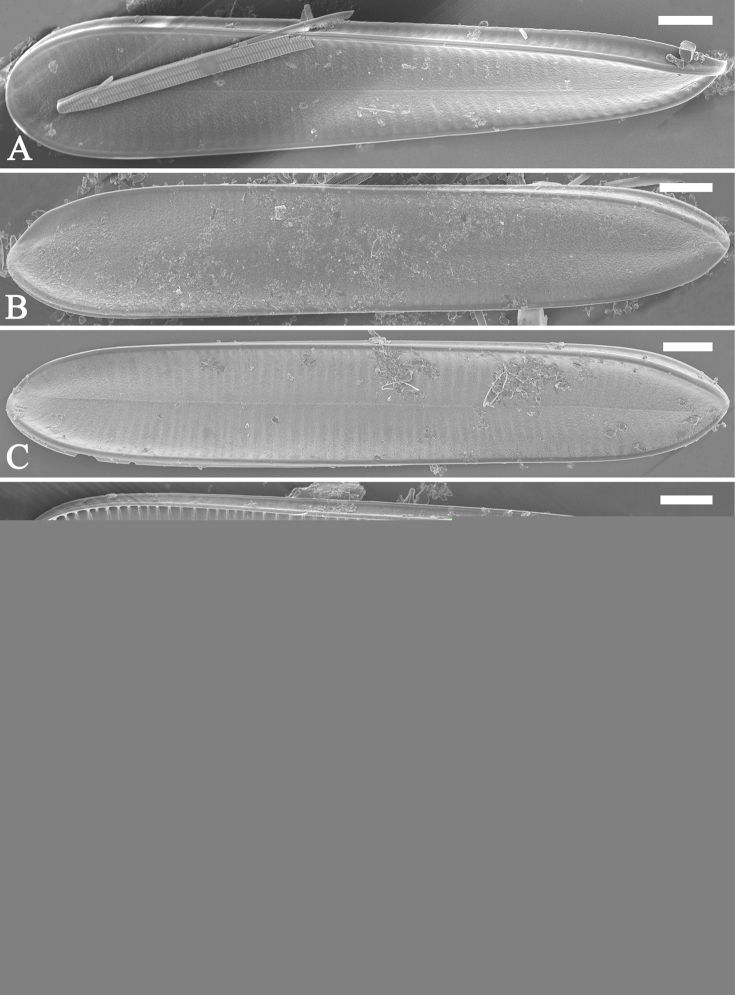
*Surirellawufluminensis* sp. nov., SEM**A–C** three valves in external view; note the visual discrepancies of twist degrees between them as the position of each valve relative to the observer differs **D–F** three valves in internal view; note the visual discrepancies of twist degrees between them. Scale bars: 20 μm.

***LM*** (Fig. 1). Valves twisted, sometimes exhibiting linear-lanceolate valve outline (Fig. 1D), with twisted and deflected apices (Fig. 1A–C). Valve dimensions (n = 27): 198–295 μm long, 41–50 μm wide. Valve face smooth without undulations, valve midline sigmoid, fibulae very distinctive, 20–28 in 100 μm. The degree of rotation (twist) of the valves differs (Fig. 1A–D) as each position relative to the observer is different. Two type valves are observed under LM: one is counterclockwise twisted (Fig. 1A, C), the other is clockwise twisted (Fig. 1B, D), also seen in SEM (Fig. 2A–F).

**Figure 3.**
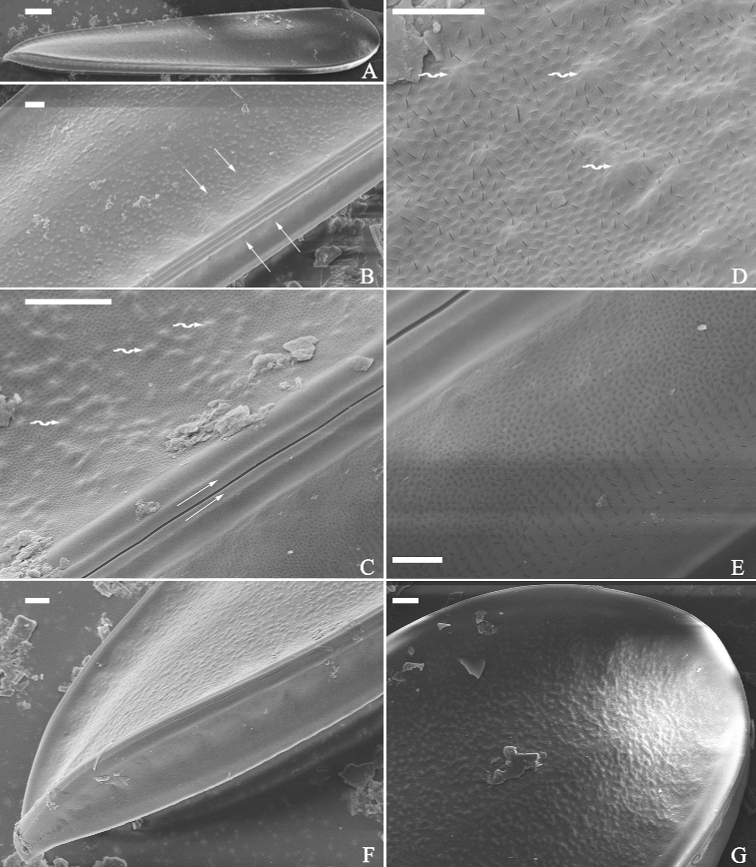
*Surirellawufluminensis* sp. nov., external view, SEM**A** a whole valve **B** middle detail from **A** showing the shallow depressions on the valve face correspond to the ones on the mantle (four arrows) **C–E** details from Fig. B showing mound-like siliceous outgrowths on the valve surface (**C, D**, three wavy arrows respectively), raised longitudinal ridges on both sides of the raphe (**C**, two arrows), slit-like external openings of areolae on both valve face and mantle (**D–F**) **F, G** apical details from **A**, note interrupted terminal raphe endings (see also Fig. 4D). Scale bars: 20 μm (**A**); 3 μm (**B, C, F, G**); 1 μm (**D, E**).

**Figure 4.**
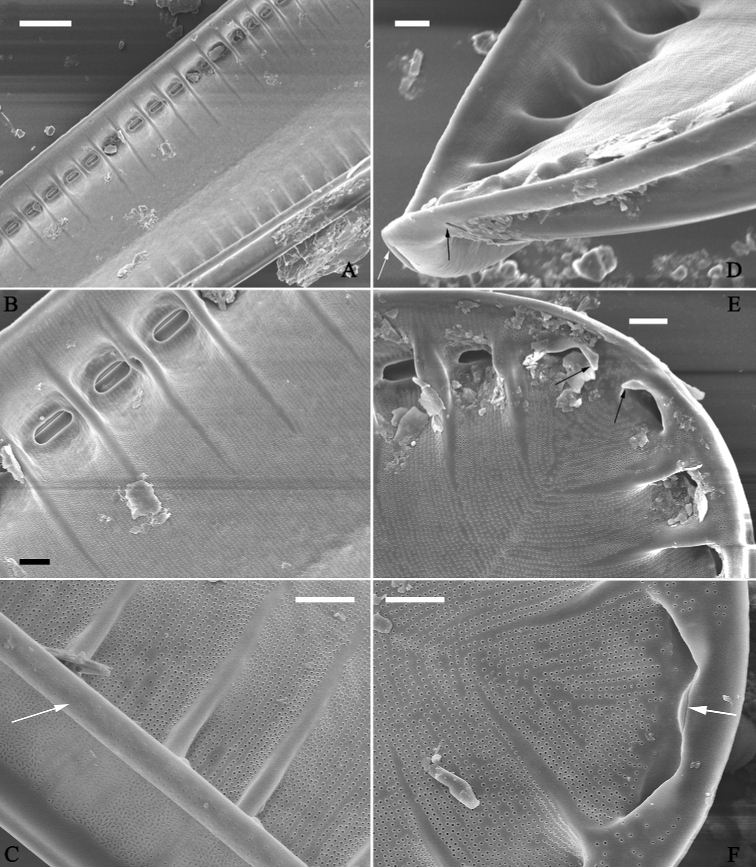
*Surirellawufluminensis* sp. nov., internal view, SEM**A–C** middle details from Fig. 2D, note that fibulae extend 1/3 to 1/2 of half valve width (**A**), one portula between two adjacent fibulae (**A, B**), uniseriate striae, and valve mantle with thickened base margin (**C**, white arrow) **D, E** apical details from Fig. 2D, note two interrupted external terminal raphe endings (**D**, two arrows), two helictoglossa-like processes (**E**, two arrows) **F** another apex showing continuous raphe at one apex (white arrow). Scale bars: 5 μm (**A**); 2 μm (**B–F**).

***SEM*** (Figs 2–4). Valve twisted, but visual discrepancies of degree of twist differs (Fig. 2A–F). Valve surface flat without undulations but mound-like siliceous outgrowths scattered throughout (Figs 2A–C, 3A–D). Very shallow depressions close to mantle, corresponding to beneath, internal fibulae (Fig. 3B, four arrows). Raised longitudinal ridges produced on both sides of raphe (Fig. 3C). Externally, costae (thickened silica ribs) absent, external openings of areolae slit-like on both valve face and mantle (Fig. 3D, E). External distal raphe endings interrupted at both apices (Figs 3F, G, 4D). Internally, distal raphe endings interrupted only at one apex where two helictoglossa-like processes present (Fig. 4E), raphe continuous at another apex (Fig. 4F). Mantle with same striation patterns as valve face and its base margin thickened (Figs 3E, F, 4C). Fibulae distinctive, extending from mantle, spanning 1/3 to 1/2 of half valve width except near two apices where fibulae meeting at midline (Figs 2D–F, 4A). Uniseriate striae 40–42 in 10 μm (measured in SEM images, n = 3). Internal openings of areolae rounded, rimmed (Fig. 4C, F). One portula and ca. 15–23 uniseriate striae located between two adjacent fibulae (Fig. 4A, B).

###### Etymology.

Named after Wu River, where the species was found.

###### Ecology and distribution.

Epilithic in a mountain river with oligotrophic waters. The following environmental parameters were measured in the field. Conductivity was 99.7 ± 0.3 μS∙cm^–1^, pH was 7.9 ± 0.1 and water temperature was 13.2 ± 0.2 °C.

##### ﻿ Surirella suiningensis

Taxon classificationPlantaeSurirellalesSurirellaceae

Bing Liu & D.M. Williams sp. nov.

1C8F581F-CFCE-50B3-89E4-441817CD6A05

Figs 5 , 6 , 7 , 8

###### Holotype.

**Figure 5.**
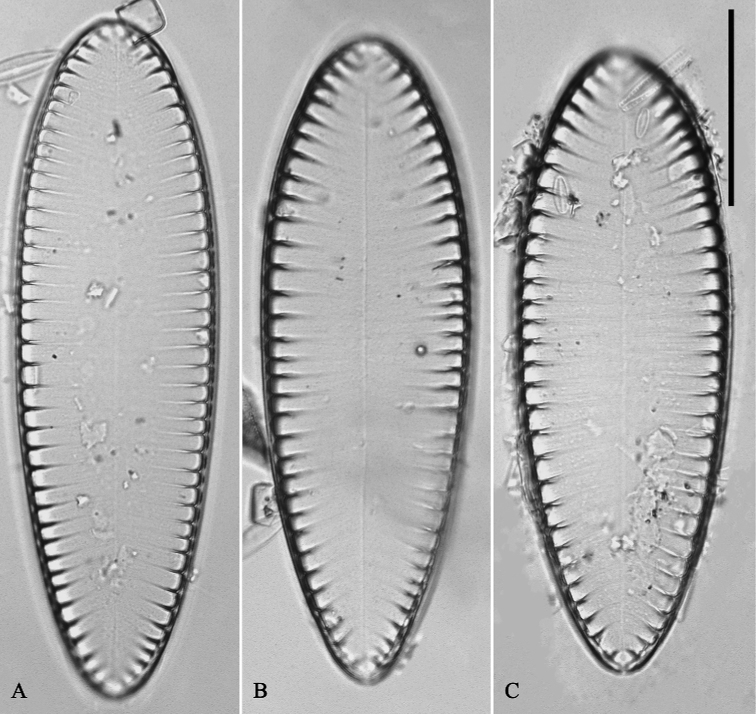
*Surirellasuiningensis*, sp. nov., LM. ×400 **A–C** three valves showing narrow-ovate outline, slightly heteropolar valves, straight valve median line, downward deflecting head and foot poles, distinctive fibulae spanning 70–80% of half valve width except near each apex where a few fibulae meet at median line. Micrograph of holotype (**B**). Scale bar: 50 μm.

***Holotype***BM! 81893, specimen circled on slide, illustrated as Fig. 5B; isotype JIU! G202107, specimen circled on slide, illustrated as Fig. 5A.

###### Type locality.

China. Hunan Province: the course of Wu River, a sampling point at Changpu Town, Suining County, 26°34.59'N, 110°09.19'E, 300 m a.s.l., collected by Bing Liu, March 22, 2021.

###### Description.

***LM*** (Fig. 5). Valves narrowly ovate, heteropolar, with downward deflecting head and foot poles; headpole broadly rounded whereas footpole narrowly rounded. Valve dimensions (n = 8): 148–173 μm long, 49–55 μm wide (measured at widest valve part). Valve face appearing smooth, without undulations and costae. Valve midline straight, downward deflecting at each apex. Fibulae very distinctive, parallel in valve middle part, radiate at two apices, 20–24 in 100 μm. Fibulae extending from mantle towards midline, spanning 70–80% of half valve width except near each apex where few fibulae nearly meet at median line.

**Figure 6.**
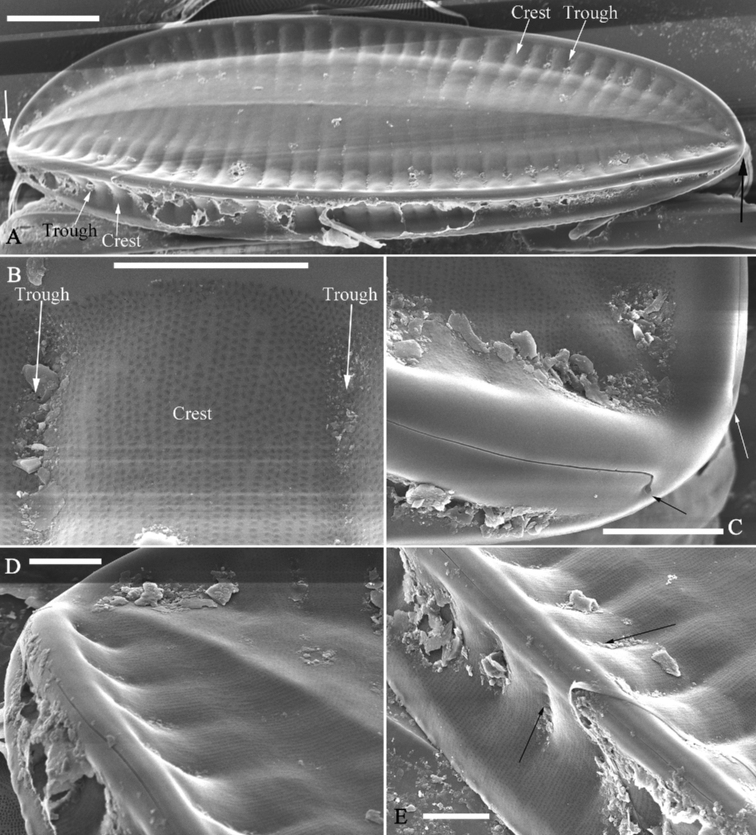
*Surirellasuiningensis* sp. nov., external view, SEM**A** one valve showing distinct continuous siliceous median ridge, downward deflecting head and foot poles (two arrows), and pattern of troughs alternating with crests **B** detail from **A** showing two troughs and one crest **C, D** apical details from **A**, note two curved terminal raphe fissures (**C**, two arrows) **E** marginal detail of **A**, note the recessed valve mantle with thickened base margin, no fenestrae existed, troughs on valve surface corresponding to those on the mantle (two black arrows). Scale bars: 20 μm (**A**); 2 μm (**B–E**).

**Figure 7.**
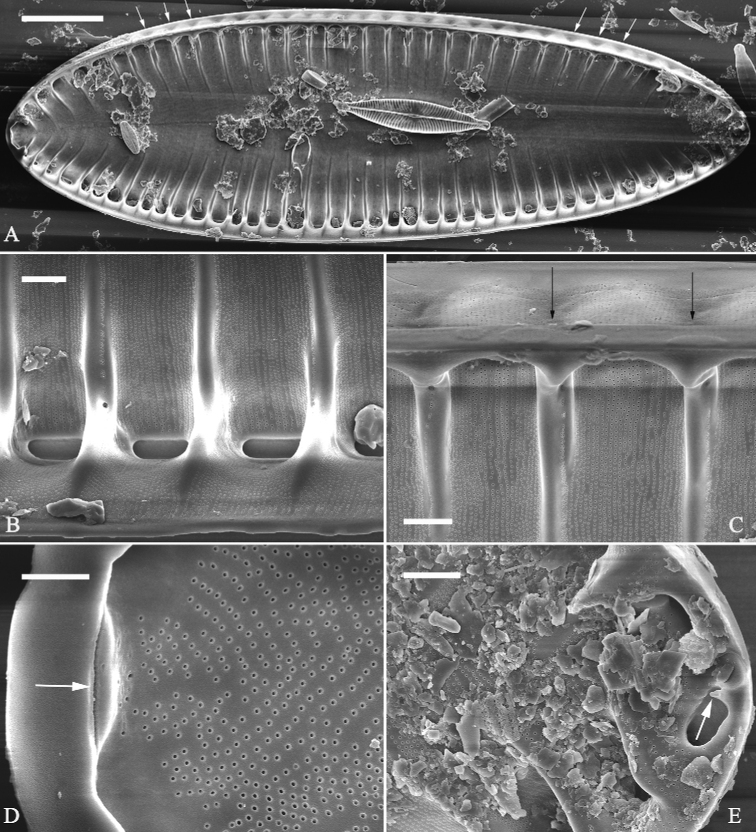
*Surirellasuiningensis*, sp. nov., internal view, SEM**A** one complete valve showing fibulae extending close to median line and fibulae corresponding to the sinking of mantle (i.e. troughs, six arrows) **B** detail from **A** showing only one portula between two adjacent fibulae and uniseriate striae **C** sinking of mantle against the fibulae (two arrows) **D, E** apical details from **B**, note the raphe continuous at one apex (**D**, arrow) whereas interrupted at the other apex (**E**, one arrow pointing at a helictoglossa-like process). Scale bars: 20 μm (**A**); 2 μm (**B, C, E**); 1 μm (**D**).

**Figure 8.**
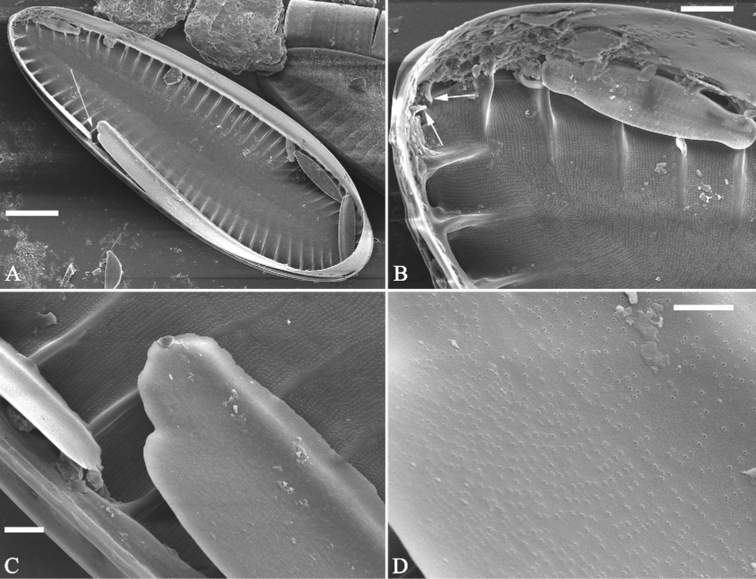
**A–D***Surirellasuiningensis*, sp. nov., internal view, SEM**A** one valve with valvocopula, note the open nature of valvocopula (arrow) **B** apical detail from **A**, note two helictoglossa-like processes (two arrows) **C** detail of open part of valvocopula **D** detail of valvocopula showing the rimmed areolae internally. Scale bars: 20 μm (**A**); 4 μm (**B**); 1 μm (**C, D**).

***SEM*** (Figs 6–8). Valve smooth, canal raphe system located around valve margins, slightly raised (Fig. 6A). Median line slightly raised, continuous from pole to pole, downward deflecting at both apices (Fig. 6A). Valve surface producing shallow troughs and higher crests (Fig. 6A, B), and troughs alternating with crests from pole to pole. Costae (thickened siliceous ribs) absent (Fig. 6B). Each trough corresponding to its internal fibula, each crest composed of c. 16–27 uniseriate striae (Fig. 6B, E). Distal raphe endings curved (Fig. 6C, D). Mantle with same pattern as valve surface, troughs align with each other (Fig. 6E). Internally, fibulae evident, extending from mantle to median line, spanning 70–80% of half valve width. Striae uniseriate, 47–51 in 10 μm. External openings of areolae slit-like (Fig. 6B) while internal openings rounded and rimmed (Fig. 7D). One portula and 16–27 uniseriate striae located between two adjacent fibulae (Fig. 7B). Sinking of mantle against fibulae present (Fig. 7C). Raphe continuous at one apex whereas interrupted at other apex (Figs 7E, 8B). Valvocopula open (Fig. 8A, C) and rimmed areolae internally (Fig. 8D).

###### Etymology.

Named after Suining County of Hunan Province, where the species was found.

###### Ecology and distribution.

Epilithic in a mountain river with oligotrophic waters. *Surirellasuiningensis* was found in the same sampling site with *S.wufluminensis*, for the environmental parameters, see above.

## ﻿Discussion

The structure of the valves in the two new species argues for their placement in the genus *Surirella*, in the sense of Ruck et al. (2016). Both have direct communication between the raphe opening and the valve interior via simple portulae, a feature recognized by Ruck et al. (2016) as a synapomorphy for the genus. The structure of the raphe, which is discontinuous in both taxa, is akin to species in the Pinnatae group (= sensu stricto group) of *Surirella* (Ruck and Kociolek 2004; Ruck et al. 2016), suggesting they might be more closely related to species in that group than species in *Cymatoleura* W. Smith. The distinct presence of an internal helictoglossa-like process at the raphe terminations in the two species can be considered as a differentiating character from other “typical” *Surirella* species, which has, so far, been reported only in a few species such as *Surirellarobusta* Ehrenberg, *S.splendida* (Ehrenberg) Kützing, *S.rumrichorum* Metzeltin & Lange-Bertalot (1998, figs 216: 2, 3; 219: 7), and *S.hinziae* Cvetkoska, Levkov & P.B. Hamilton (in Cvetkoska et al. 2015, p. 187, fig. 32).

*Surirellaaquastudia*, *S.xinjiangiana*, and *S.dongtingensis* all have twisted frustules and all produce undulations on the valve surface from pole to pole. Previously, based on the presence of valve undulations, they would have been placed in the genus *Cymatopleura*. *Surirellawufluminensis* on the other hand, lacks undulations on its valve surface. Other similar species demonstrating valves with torsion include the former *Surirellaspiralis* Kützing (Wang 2018) – this species is now recognized as a member of *Iconella*, as *I.spiralis* (Kützing) Ruck & T. Nakov. The only taxa similar to *S.wufluminensis*, in the sense of having twisted valves and lacking central valve undulations, are *S.subcontorta* Hustedt (in Schmidt 1942, plate 356, figs 1, 2), described from the African Rift Lake of Tanganyika, and *S.uninodes*Skvortzov (1937, p. 360) from Lake Baikal. *Surirellasubcontorta*, as illustrated and described by (Hustedt 1942) is much wider at the ‘headpole’ than *S.wufluminensis*. *Surirellauninodes* is twisted near the middle of the valve, quite unlike *S.wufluminensis* (Hustedt 1942).

With regards to *S.suiningensis*, the ovate valve outline is reminiscent of *Surirelladavidsonii* A.W.F. Schmidt, *S.elegans* Ehrenberg, *S.slesvicensis* Grunow (in Schmidt 1875, plate 21, fig. 19) as well as *Iconellaguatimalensis* (Ehrenberg) Ruck & Nakov. *Surirellasuiningensis* has narrow-ovate valve outline (i.e., its headpole is only slightly wider than its footpole) while *S.davidsonii* has an ovate valve outline (i.e. its headpole is wider than its footpole). SEM images of *S.davidsonii* have been published by Metzeltin and Lange-Bertalot (1998). *Surirellaelegans* produces a lanceolate central region which *S.suiningensis* lacks. *Surirellaslesvicensis*, described from a swamp in Europe, is similar to *S.suiningensis* in shape and overall proportions. De Toni (1892) suggested *S.slesvicensis* may be conspecific with *S.elegans* and *S.subalpina*Donkin (1869, p. 292) described from the U.K. There are no records of *S.slesvicensis* or *S.subalpina* being studied with electron microscopy (Gaul et al. 1993; Henderson and Reimer 2003).

*Surirellawufluminensis* and *S.suiningensis* occur together in the same freshwater habitat. Their associated species comprise *Pinnulariahustedtii* Meister (see Williams et al. 2022, p. 294), *Diatomavulgaris*Bory de Saint-Vincent (1824, p. 461), *Tabulariafonticola* (Hustedt) C.E. Wetzel & D.M. Williams (in Vigneshwaran et al. 2020, p. 179), and some species of *Fragilaria*Lyngbye (1819, p. 182), *Gyrosigma*Hassall (1845, p. 435), *Navicula*Bory de Saint-Vincent (1822, p. 128), *Nitzschia*Hassall (1845, p. 435), *Sellaphora*Mereschkowsky (1902, p. 186) and among others.

## Supplementary Material

XML Treatment for Surirella wufluminensis

XML Treatment for Surirella suiningensis
